# Bone defect repair on the alveolar wall of the maxillary sinus using collagen membranes and temporal fascia. An experimental study in monkeys

**DOI:** 10.1590/S1808-86942011000400006

**Published:** 2015-10-19

**Authors:** Adalberto Novaes Silva, José Américo de Oliveira, Maria Célia Jamur, José Ari Gualberto Junqueira, Vani Maria Correa, Wilma Terezinha Anselmo Lima

**Affiliations:** 1 PhD, assistant professor; 2 PhD, assistant professor; 3 PhD, full professor; 4 Technician; 5 Technician; 6 Associate professor, faculty member

**Keywords:** bone regeneration, collagen, fascia, maxillary sinus, membranes

## Abstract

**Abstract:**

Few studies has been done using guided bone regeneration in maxillary sinus defects.

**Aim:**

To assess the bone repair process in surgical defects on the alveolar wall of the monkey maxillary sinus, which communicates with the sinus cavity, by using collagen membranes: Gen-derm - Genius Baumer, Pro-tape - Proline and autologous temporal fascia.

**Materials and Methods:**

In this prospective and experimental study, orosinusal communications were performed in four tufted capuchin monkeys (Cebus apella) and histologic analysis was carried out 180 days after.

**Results:**

In the defects without a cover (control), bone proliferation predominated in two animals and fibrous connective tissue predominated in the other two. In defects repaired with a temporal fascia flap, fibrous connective tissue predominated in three animals and bone proliferation predominated in one. In the defects repaired with Gen-derm or Pro-tape collagen membranes there was complete bone proliferation in three animals and fibrous connective tissue in one.

**Conclusions:**

Surgical defect can be repaired with both bone tissue and fibrous connective tissue in all study groups; collagen membranes was more beneficial in the bone repair process than temporal fascia or absence of a barrier.

## INTRODUCTION

Defects on the wall of the maxillary sinus that result in buccal-sinus communications occur for several reasons. They may be secondary to dental diseases (such as tooth extractions or implant surgery), chronic periodontal disease, endodontic treatment, orthognathic surgery, facial trauma, surgery for neoplasms that involve the maxilla and maxillary sinus, sinus infections, and otorhinolaryngologic surgical procedures^1^, ^2^, ^3^, ^4^, ^5^.

If the maxillary sinus is perforated traumatically and a buccal-sinus communication ensues (such as after accidental perforation while extracting a posterior upper tooth), it is thought that the bone defect may heal with neoformed bone or a fibrous union, resulting in mucoperiosteal closure of the mouth and sinus^6,^^7^. It may also progress to non-union, resulting in a fistula. This occurs when the communication between the mouth and the maxillary sinus becomes lined by epithelial tissue due to proliferation from adjacent tissues to the bone defect^8^.

Successful treatment depends on the duration of the communication, absence of prior sinus disease, the size and site of the defect, and the quantity and quality of adjacent tissues^9^. Several studies have underlined the importance of early closure of oroantral holes. Once an oroantral communication is established, treatment should begin as early as possible, because changes in the mucosa may cause sinus diseases (infection)^7^.

The treatment of buccal-sinus communications consists of local or distance flaps and interposed autogenous grafts or alloplastic implants. Local flaps are mainly oral vestibule or palatal tissues^10,^^11^. Other reported possibilities are buccinator myomucous^12^ and tongue^13^ flaps. Many authors have recommended closing the fistula in two levels^14^, ^15^, ^16^, ^17^, ^18^, ^19^. Treatment of the buccal-sinus communication is considered successful if soft tissue (mucoperiosteum) on the oral side is closed. Little has been reported on repair of the bone defect in a treated buccal-sinus communication where there is neoformed bone or fibrous connective tissue, as well as the quality of these tissues.

Another possibility for the treatment of buccal-sinus communication is to apply the principles of guided bone regeneration^20^ in which a microporous membrane is used to allow desired cells to invade a clot and to stop undesired cells reaching the bone defect, thereby isolating the area to be repaired from the surrounding epithelial and loose connective tissue. This technique mechanically stops soft tissues from invaginating into the surgical defect, which allows the organism to neoform bone tissue. Guided bone regeneration aims to reduce the bone regeneration time and provide better quality neoformed bone.

Because of the possibility of using esthetic and functional dental implants, it is currently important to take into account the bone viability in the buccal-sinus communication; if the patient eventually requires osteointegrated implants, sufficient quality bone will be required for successful integration^21^. As there have been many studies on guided bone regeneration in periodontics and implantdontics, contrasting with few studies that have applied this principle in the repair of maxillary sinus bone perforations ^7,^^20,^^22,^^23^, out aim was to investigate whether guided bone regeneration could shorten the bone regeneration time and yield quality bone for placing implants in the site of a buccal-sinus communication.

Several materials have been used in guided bone regeneration. Collagen-derived products are widely used 23, 24, 25. There have been few studies on the use of temporal fascia in guided bone regeneration^26,^^27^.

Thus, the purpose of our study was to carry out a histological evaluation of maxillary sinus alveolar wall repair of a communication with the sinus, and to compare qualitatively the repair process with three guided bone regeneration barrier materials: Pro-tape collagen membrane (Proline), Gen-derm collagen membrane (Genius Baumer), and autologous temporal fascia.

We believe that the anatomical similarity between the maxillary sinus of the tufted capuchin monkey (*Cebus apella*) and that of humans provides an appropriate experimental model for studying these surgical approaches.

## MATERIALS AND METHODS

This study was approved by the Tufted Capuchin Monkey Procreation Center, Basic Science Department, Araçatuba Dentistry School - UNESP (FOA no. 087/95) and the institutional review board of the Clinic Hospital, Ribeirão Preto Medical School, São Paulo University (Protocol no. 1930/97). Four *Cebus apella* monkeys were used in this study. The animals were observed for 40 days before starting the experiment. The monkeys were given food and water without restrictions before and after surgery, except for the first postoperative week, when they were given a liquid diet (Gatorade and fruit yogurt). The animals were operated and kept in individual cages throughout the experiment. The mean weight of the animals was 2.4 kg; they were aged between 6 and 7 years, therefore considered young adults in Gilmore's criteria^28^.

In each surgical procedure, the animals were sedated by sulphur ether inhalation in an appropriate chamber. Next, a benzodiazepine agent was injected intramuscularly (diazepam - 0.3 mg/kg), and a barbiturate (sodium thiopental 30 mg/kg) was injected intraperitoneally. A 2% xylocaine/epinephrine (1:200.000) solution was injected with a carpule syringe in the maxillary operation sites before starting the procedure. Sterilized surgical drapes and materials were used for surgical asepsis.

Each animal was placed on lateral decubitus over a surgical drape. Antisepsis of the face was done with alcohol iodine; the mouth was cleaned with a 0.12% chlorhexidine solution in gauze. The body of the animal was then fully covered with a sterile surgical drape to expose only the face. Any dental calculi were removed before the surgical incision. Careful suction of the oropharynx was done to avoid aspiration of secretions and blood into lower airways as the animals were not intubated. A mouth opener was placed between the canine teeth on the contralateral side to the operation site.

**Figure 1:**
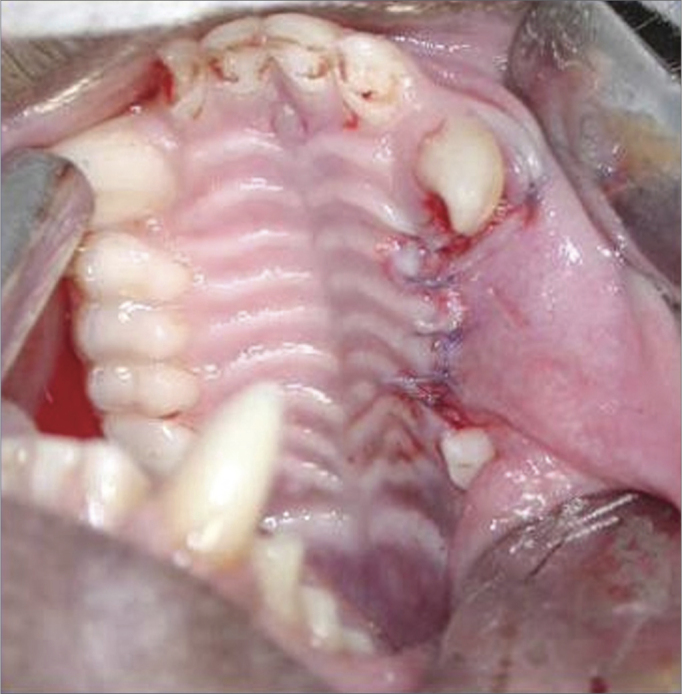
Exodontia of three upper premolars.

The three upper premolar teeth were extracted bilaterally in the first surgical phase (Fig. 1). For teeth removal, the need for vestibular osteotomies (partial alveolectomies) was checked using no. 702 surgical drills at high rotation. Constant and abundant irrigation was done with 0.9% saline. Surgical exposure was attained with interpapillary and intrasulcular incisions from the first upper premolar (P1) to the third upper premolar (P3), as well as two 0.5 cm relaxing incisions mesial to P1 and distal to P3, to facilitate mucoperiosteal detachment. Exodontia was done with human pediatric levers and forceps. A periosteal incision and flap was done to attain occlusive closure. Absorbable no. 4.0 polyglactin 910 sutures were used.

Animals were given antibiotics (100 mg/kg intramuscular cephalotin) in the immediate pre- and postoperative periods. Each animal was given 0.5 ml diclofenac and 0.5 ml sodium dipyrone intramuscularly at the end of surgery. Upon awakening, animals were also given acetaminophen drops in the diet for 48 hours for postoperative analgesia.

**Figure 2:**
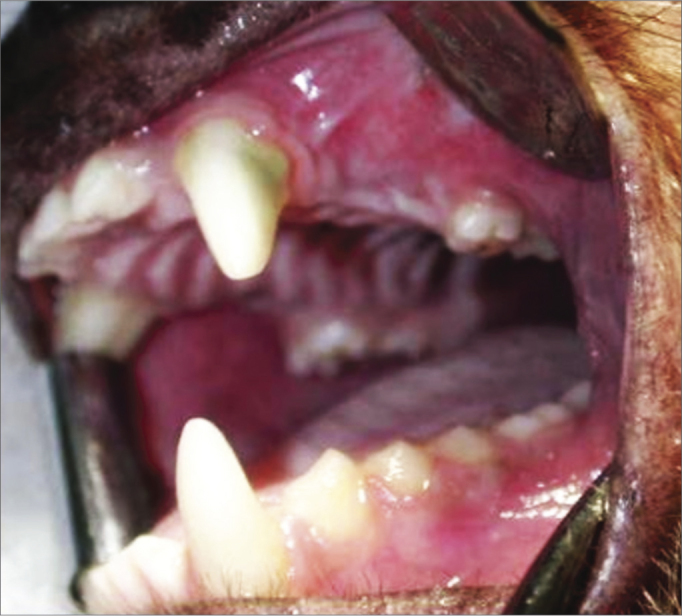
Three months after the first surgery - alveolar repair phase.

**Figure 3:**
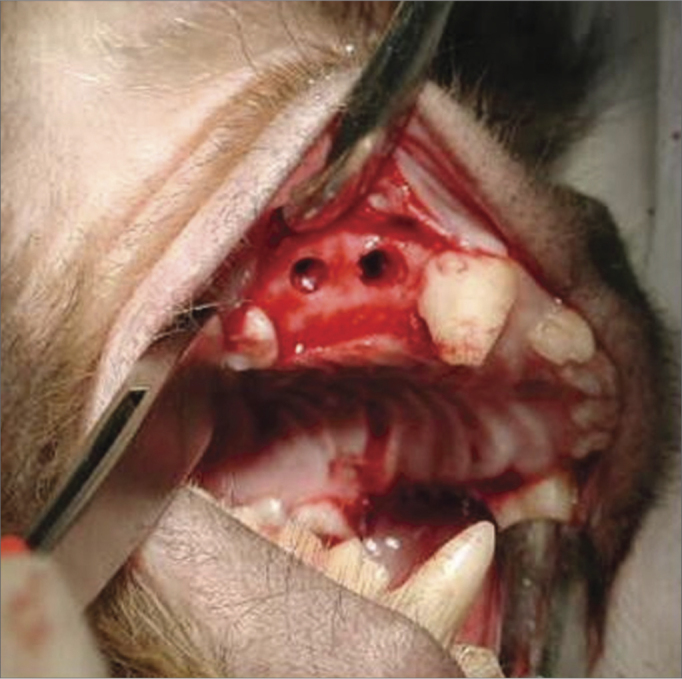
Perforations communicating with the maxillary sinus.

**Figure 4:**
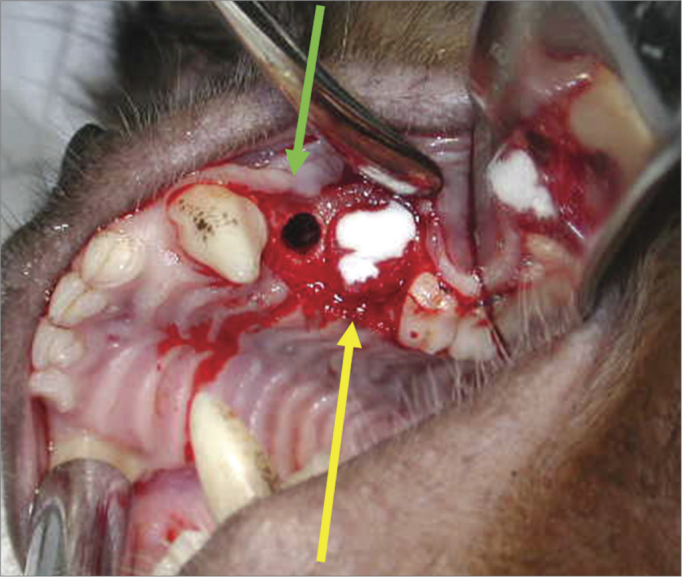
Control surgical defect (green arrow); Surgical defect with a collagen membrane (yellow arrow).

**Figure 5:**
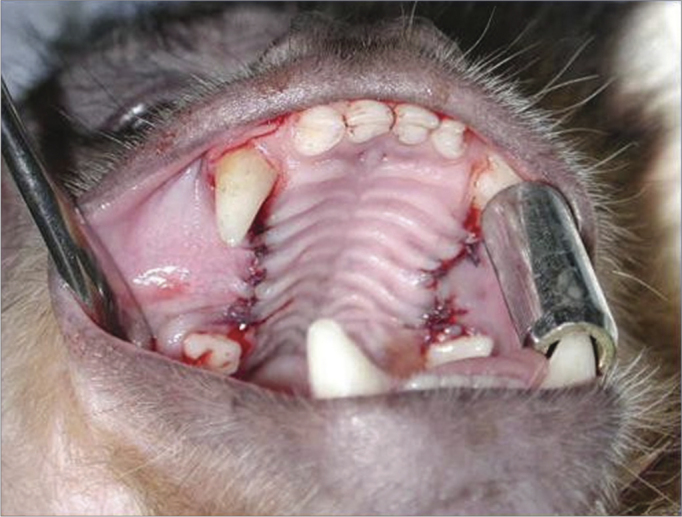
Repositioned and sutured mucoperiosteal flap.

A 90-day period was allowed for the alveolar repair process to become established (Fig. 2). At this point, the animals were again anesthetized as described above for the second surgical procedure - the same anesthesia and antiseptic procedures as above were applied. In this second phase, an incision was done on the alveolar ridge from which the teeth had been removed (from mesial P1 to distal P3), and the mucoperiosteum was detached along the gingivojugal sulcus to expose the bone tissue on the crest of alveolar ridge. Two standard diameter perforations were made with a 3.3 mm implant drill on the alveolar bone wall of the maxillary sinus on each side of the maxilla, including the sinus mucosa and penetrating into the maxillary antrum (Fig. 3). The bone thickness from the alveolar ridge surface to the maxillary floor ranged from 3 to 4 mm. An anterior perforation was done (distal to the upper canine) followed by a posterior perforation (mesial to the first upper molar). The left anterior perforation of the maxilla was the control; no guided bone regeneration was used in this perforation. The left posterior perforation was covered with a Pro-tape (Proline) collagen membrane barrier. The right anterior perforation was covered with a Gen-derm (Genius Baumer) collagen membrane barrier. The posterior perforation was covered with a temporal fascia barrier taken from the animal itself. The diameter of barrier materials was about 6 mm, which was enough to cover the perforation borders (Fig. 4). The flaps were approximated with #4.0 polyglactin 910 sutures (Fig. 5). The alveolar ridge incisions were made slightly palatal such that the sutures did not remain over the bone perforations when the flaps were repositioned. The temporal fascia fragments were harvested by making a small incision anterior to the ear and superior to the zygomatic arch on the right of the animal's face; the skin was closed with #5.0 nylon sutures. Postoperative medication and care was the same as in the first surgery.

The animals were sedated and anesthetized as described above on the 180^th^ day after the second procedure. Complete tissue fixation was attained by perfusion through the ascending aorta according to a systemic perfusion protocol.

The animals were sacrificed, and the right and left maxillae were removed and fixed in a 10% formalin solution in 0.1 molar phosphate buffer. Decalcification was done using a 10% trichloroacetic acid solution for 60 days.

At the end of decalcification, the specimens were treated routinely for paraffin inclusion to obtain 6 μm thickness histologic sections. Frontal plane semi-serial sections at 60 μm intervals were made from the distal portion of the upper canine and the mesial portion of the first upper molar. Thirty sections were made for each experimental defect; these sections were stained with hematoxylin-eosin and picrosirius red. Qualitative histology was done with conventional optic and polarized light microscopy.

## RESULTS

No sign of bucal-sinus fistulae or changes in the mucosal surface were seen clinically at the moment the animals were sacrificed.

Proliferated bone completely filled in the control experimental defects in which no barriers were applied (bone perforations were not covered with membranes) in two animals; fibrous connective tissue was present in the surgical defect of the other two animals. There was complete bone neoformation in the operated sites of three animals with experimental defects in which Pro-tape and Gen-derm collagen membranes were applied; connective fibrous tissue was present between the bone ridges in one animal. The operated sites were completely filled by neoformed bone in one animal in which a temporal fascia barrier was placed over the experimental defect; fibrous connective tissue was present in the surgical defect in three animals (Table 1).

**Table 1:** List of experimental surgical defects (control, with collagen membranes Pro-tape, Gen-derm, and temporal fascia) and the type of repair (completely neoformed bone, and fibrous connective tissue).

	Repair with completely neoformed bone	Repair with fibrous connective tissue
Experimental defect without barrier	2 animals	2 animals
Experimental defect with Pro-tape membrane	3 animals	1 animal
Experimental defect with Gen-derm membrane	3 animals	1 animal
Experimental defect with autogenous temporal fascia	1 animal	3 animals

**Figure 6:**
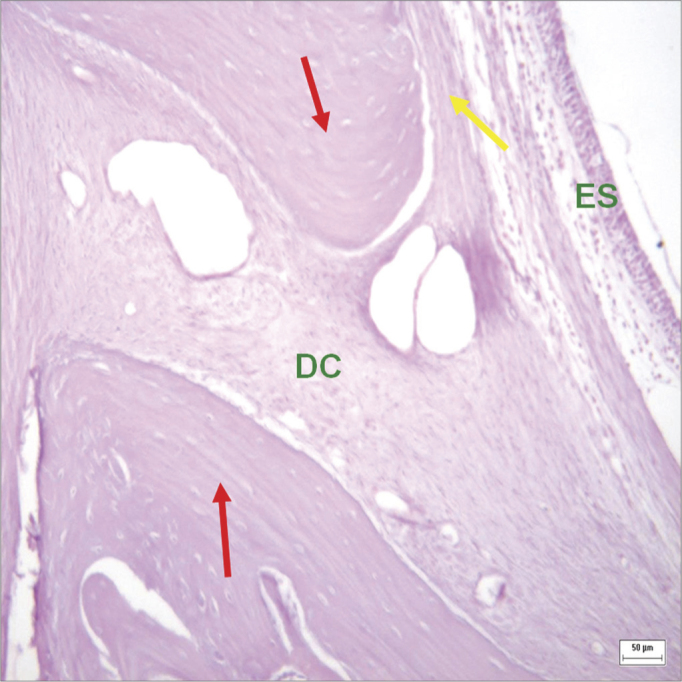
Histological section of an experimental surgical defect not completely filled with bone (hematoxylin-eosin stained) - bone riddges (red arrows); surgical defect (DC) containinig fibrous connective tissue; periosteium (yellow arrow); sinus epithelium (ES).

**Figure 7:**
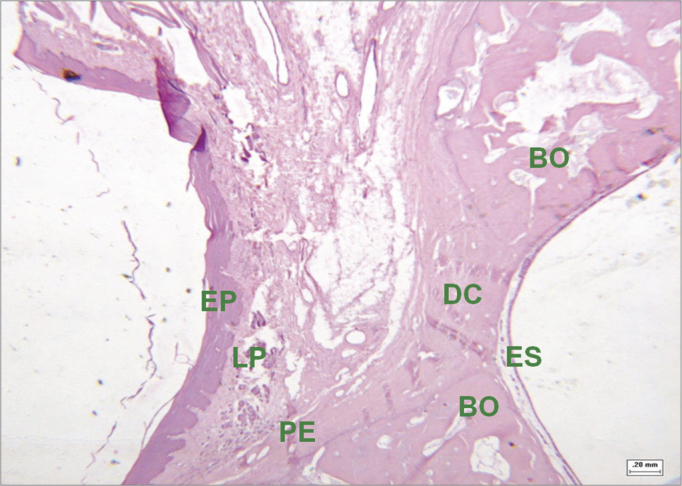
Histological section of an experimental surgical defect not completely filled with bone (hematoxylin-eosin stained) - surgical defect (DC) containing fibrous connective tissue; squamous epithelium (EP); lamina propria (LP); bone ridges (BO); periosteum (PE); sinus epithelium (ES).

Connective tissues within bone ridges consisted of dense bundles separating the oral mucoperiosteum from the sinus mucoperiosteum in experimental defects that were not completely filled in by bone. In these areas, collagen fibers were parallel; no bone formation islands were seen within the fibrous tissues. (Figs. 6 and 7).

Fibrous connective tissues filled all of the experimental surgical defects (control, with Gen-derm and Pro-tape collagen membranes, and with temporal fascia) in one animal.

**Figure 8:**
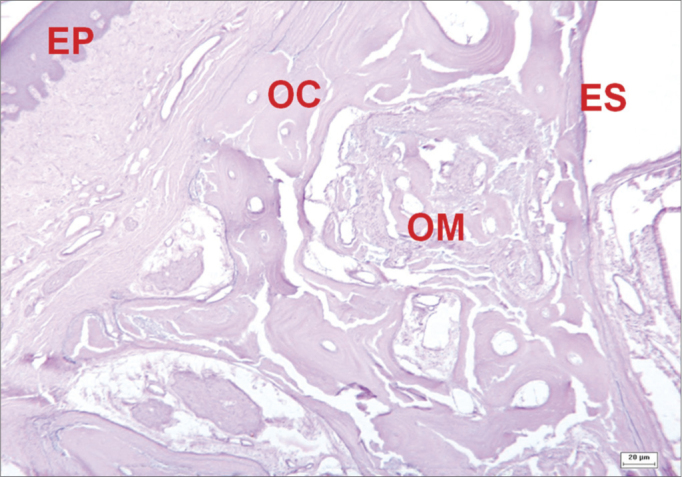
Histological section of an experimental surgical defect with no bone discontinuity, showing bone regeneration sites (hematoxylin-eosin stained). Compact bone (OC); medullary bone (OM); squamous epithelium (EP); sinus epithelium (ES).

Bone walls regenerated in the operated sites of animals in which surgical defects were completely filled in with bone; in these cases, the transition between neoformed and adjacent bone was difficult to identify. These areas contained well-defined bone trabeculae, at times with formed Haversian systems. Bone regeneration areas with abundant vascularity and embryonic-type tissue with mesenchymal cells was identified in a few fields (Fig. 8). There were also areas containing osteoblasts in rows next to the bone matrix trabeculae; their cytoplasm was strongly basophilic, indicating protein synthesis - mainly type I collagen.

Sinus mucosa regeneration was constantly encountered after the 180^th^ postoperative day. These fields had no inflammatory tissues. Remains of the barrier materials (collagen membranes and temporal fascia) for guided bone regeneration were also not seen.

In all study groups (no barrier, Pro-tape membrane, Gen-derm membrane, and temporal fascia), the experimental surgical defects that had been filled with neoformed bone were not significantly different histologically.

**Figure 9:**
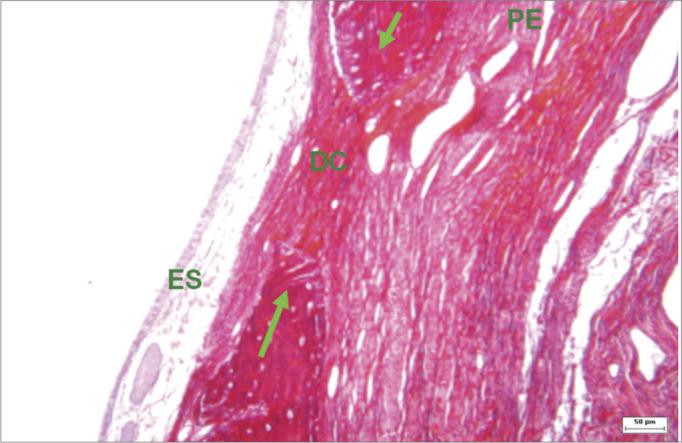
Histological section of an experimental surgical defect not completely filled in by bone (picro sirius red stained), under clear field microscopy - fibrous connective tissue in the surgical defect (DC), where red, orange, and yellow fibers predominate, suggesting type I collagen; sinus epithelium (ES); periosteum (PE); bone ridges (green arrows).

**Figure 10:**
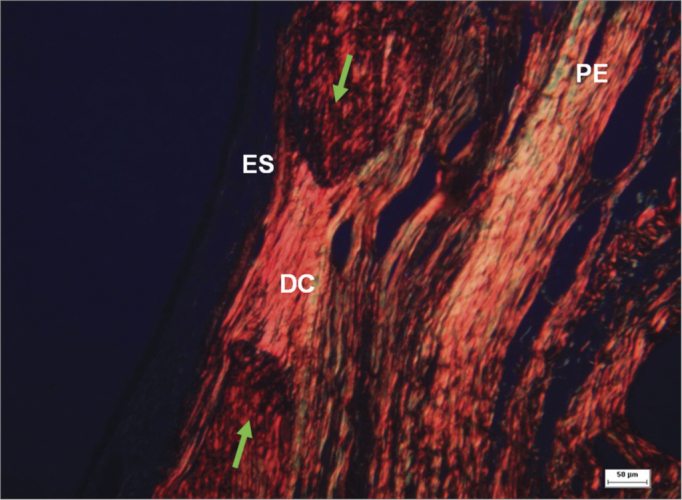
Histological section of an experimental surgical defect not completely filled in by bone (picro sirius red stained), under polarized light microscopy - surgical defect (DC) showing dense and parallel collagen bundles with red, orange, and yellow birefringent fibers, suggesting the presence of type I collagen; sinus epithelium (ES); periosteum (PE); bone ridges (green arrows).

We found organized and distinct collagen fibers (picrosirius red stained and seen under clear field and polarized light microscopy) 180 days after surgery in the groups where fibrous connective tissue remained in the bone perforation site. Clear field microscopy showed in red the collagen in surgical defects that were not filled completely by bone (Fig. 9). Polarized light microscopy revealed dense parallel collagen bundles - red, orange, and yellow birefringent fibers - suggesting type I collagen (Fig. 10). Collagen fibers were also located in connective tissues of the lamina propria, forming a layer below the ketatinized squamous stratified epithelium.

## DISCUSSION

Neto & Volpon^29^ have suggested that although fibrous union is a frequent complication of bone surgery, it is not easy to replicate experimentally in animals. From biological and clinical perspectives, it is preferable for bone defects to be repaired with bone rather than fibrous connective tissue^7^. In buccal-sinus communications, local bone regeneration in sufficient quantity and quality is essential for indicating osteointegrated implants^21^. Otherwise, conventional prostheses would have to be used to rehabilitate edentulous areas.

In our study, bone proliferated in two animals and fibrous connective tissue filled in the communication in the other two when the experimental surgical defects were not covered. The potential that periosteum has to form and regenerate bone has been demonstrated in several studies^30,^^31^. However, some authors have argued that using periosteum is limited because of its inability to preserve space^23^.

In the present study, experimental surgical defect were filled in completely by proliferated bone in three animals when Pro-tape (Proline) and Gen-derm collagen membranes were applied. Matisko et al.^7^ studied the bone repair ability in rabbit maxillary sinus bone wall defects with and without collagen membranes. Their results showed that a collagen barrier had a beneficial effect following exposure of the maxillary sinus. Dupoirieux et al.^25^ explained unsatisfactory results in guided bone regeneration with collagen membranes by the poor mechanical properties of these membranes - excess flexibility and lack of enough rigidity to maintain the space that was to be repaired.

We expected good results when using a temporal fascia barrier - an autogenous substance - to facilitate bone regeneration. However, fibrous connective tissue was present between the bone ridges of the surgical defect (separating the oral from the sinus mucoperiosteum) in three of four animals in which the experimental bone defect was covered with autogenous temporal fascia. We could not explain concretely this event. We debated that because it was fresh untreated tissue - as opposed to laboratory-processed collagen membranes - temporal fascia may have retracted in the initial repair phase of the surgical defect, and that rather than covering the margins of the perforation, it may have remained within the defect, thereby interfering with bone regeneration. Silverstein et al.^26^ reported that fascia lata is biocompatible and well tolerated in tissues, and may be a regenerative collagen membrane. Sarac & Bulent^32^ showed that homogenous temporal fascia could be applied in tympanoplasties and attain similar success to autogenous temporal fascia.

After 180 days, dense and parallel collagen bundles (picrosirius red stained and seen under polarized light microscopy) were observed in areas where fibrous connective tissue persisted within the experimental surgical defect^33^. These fibers were red, orange, and yellow birefringent, which suggested type I collagen - found mainly in bone, tendons, the dermis, and dentin. A strongly birefringent field is typical of structures with molecules aligned in a single direction - again typical of types I and III collagen. Type III collagen is green birefringent if a picrosirius red stain is used and seen under polarized light microscopy; this type was not encountered in our sample.

Haanaes & Gilhuus-Moe^6^ showed that epithelial proliferation was more pronounced in the antral wall throughout all observation periods in an experimental model of buccal-sinus communication through alveoli following molar teeth extraction in monkeys. We did not use barriers on the sinus wall of the communication because the experimental model did not allow this possibility, and because this model would not be reproducible in humans. Thus, barrier materials may have blocked fibroepithelial tissue from invading the experimentally created defect on the oral side but not the sinus side of these of perforations.

We concluded that, irrespective of the sample size (four animals), alveolar wall bone defects communicating with the maxillary sinus are best repaired by the use of collagen membranes (Gen-Derm/Pro-tape) compared to absence of these barriers; bone neoformation is even less satisfactory when autogenous temporal fascia is used.

The need to rebuild maxillary and mandibular bone defects has changed the focus of surgery in several procedures. These include more conservative exodontias together with immediate bone reconstruction, or alveolar atrophy preventive methods, use of grafts, platelet rich plasma, morphogenetic bone protein, and guided tissue regeneration. Our results demonstrate the need for further studies of treatment approaches for buccal-sinus communications to attain satisfactory repair of the bone defect and adequate morphofunctional rehabilitation of the operated site.

## CONCLUSION

The use of collagen membranes (Gen-derm/Pro-tape) for guided bone regeneration yielded significant benefits in the surgical repair of the alveolar wall (maxillary sinus communication) in this comparison of sites without barriers and sites where temporal fascia was used.

## References

[1] Heling I, Rotstein I. (1989). A persistent oronasal sinus tract of endodontic.. J Endod..

[2] Felix DH, Wray D, Smith GLF, Jones GA. (1991). **Oro-antral fistula: an un**usual complication of HIV-associated periodontal disease.. Br Dent J.

[3] Aksungur EH, Apaydin D, Gonlusen G, Kiroglu M, Soylu L, Duce MN, Cosar E. (1994). A case of oroantral fistula secondary to malignant fibrous histiocytoma.. Eur J Radiol..

[4] Morgan MK, Aldren CP. (1997). Oroantral fistula: a complication of transantral ligation of the internal maxillary artery for epistaxis.. J Laryngol Otol..

[5] Agnihotri N, Spinnato G, Ziccardi V. (2007). Oroantral communication with chronic sinusitis 8 years after maxillofacial trauma: a case report.. J N J Dent Assoc..

[6] Haanaes HR, Gilhuus-Moe O. (1972). A histologic study of experimental oro-paranasal communications in monkeys.. Int J Oral Surg..

[7] Matisko LM, Wallace JA, Mundell R, Schumtz J, Zullo T. (1999). Healing of osseous maxillary sinus defects using guided tissue regeneration: An experimental study in rabbits.. J Endod..

[8] Rezende RA, Heitz C., Zanini SA. (1990). Cirurgia e Traumatologia Buco-Maxilo-Facial..

[9] Awang M. (1988). Closure of oroantral fistula.. Int J Oral Maxillofac Surg..

[10] James R. (1980). Surgical closure of large oroantral fistula using a palatal island flap.. J Oral Surg..

[11] Killey HC, Kay LW. (1967). An analysis of 250 cases of oro-antral fistula treated by the buccal flap operation.. Oral Surg Oral Med Oral Path..

[12] Carstens MH, Stofman GM, Sotereanos GC, Hurwitz DJ. (1991). A new approach for repair of oro-antral-nasal fistulaes. The anteriorly based buccinator myomucosal island flap.. J Craniomaxfac Surg..

[13] Coghlan K, O'Regan O, Carter J. (1989). Tongue flap repair of oro-nasal fistulae in cleft palate patients.. J Craniomaxfac Surg..

[14] Quayle AA. (1981). A double flap technique for closure or oronasal and oroantral fistula.. Br J Plast Surg..

[15] Hanazawa Y, Itoh K, Mabashi T, Sato K. (1995). Closure of oroantral communications using a pedicled buccal fat pat graft.. J Oral Maxillofac Surg..

[16] Martin-Granizo R, Naval L, Costas A, Goizueta C, Rodriguez F, Monge F, Muñoz M, Diaz F. (1997). Use of buccal fat pad to repair intraoral defects: review of 30 cases.. Br J Oral Maxillofac Surg..

[17] Allais M, Maurette PE, Cortez ALV, Filho JRL, Mazonetto R. (2008). Retalho do corpo adiposo bucal no fechamento de comunicação buco-sinusal.. Rev Bras Otorrinolaringol..

[18] Poeschl PW, Baumann A, Russmueller G, Poeschl E, Klug C, Ewers R. (2009). Closure of oroantral communications with Bichat's buccal fat pad.. J Oral Maxillofac Surg..

[19] Madeira M.C., da Anatomia Face. (2003). Bases anátomo-funcionais para a prática odontológica..

[20] Waldrop TC, Semba SE. (1993). Closure of oroantral communication using guided tissue regeneration and an absorbable gelatin membrane.. J Periodontol..

[21] Smith DE, Zarb GA. (1989). Criteria for success of osseointegrated endosseous implants.. J Prosthet Dent..

[22] Becker J, Neukam F, Schliephake H. (1992). Restoration of the lateral sinus wall using a collagen type I membrane for guided tissue regeneration.. Int J Oral Maxillofac Surg..

[23] Hardwick R, Scantlebury T, Sanchez R, Whitley N, Ambruster J., Buser D, Dahling C, Schenck RK. (1994). Guided bone regeneration in implant dentistry..

[24] Bunyaratavej P, Wang HL. (2001). Collagen membranes: a review.. J Periodontol..

[25] Dupoirieux L, Pourquier D, Picot MC, Neves M. (2001). Comparative study of three different membranes for guided bone regeneration of rat cranial defects.. Int J Oral Maxillofac Surg..

[26] Silverstein LH, Kraft JD, Wand R. (1992). Bone regeneration and tissue acceptance of human fascia lata grafts adjacent to dental implants: a preliminary case report.. J. Oral Implantol..

[27] Unsal B., Kurtis B., Ozcan G., Ozdemir A, Karaoz E. (1997). An investigation of resorption and tissue reaction after subcutaneous implantation of collagen based materials in rats.. J. Marmara Univ Dent Fac..

[28] Gilmore RM. (1943). Mammalogy in an epidemiological study of jungle yellow fever in Brazil.. J Mammal..

[29] Dos Santos Neto FL, Volpon JB. (1984). Experimental nonunion in dogs.. Clin Orthop Relat Res..

[30] Reide CA, McCarthy JG, Kolber AB. (1981). A study of regeneration in parietal bone defects in rabbits.. Plast Reconstr Surg..

[31] Lemperle SM, Calhoun CJ, Curran RW, Holmes RE. (1998). Bone healing of large cranial and mandibular defects protected from soft tissue interposition: A comparative study of spontaneous bone regeneration, osteoconduction, and cancellous autografting in dogs.. Plast Reconstr Surgery..

[32] Sarac S, Bulent G. (2002). Use of homograft dehydrated temporal fascia in tympanoplasty.. Otol Neurotol..

[33] Junqueira LC, Bignolas G, Brentani R. (1979). Picroirius staining plus polarization microscopy, a specific method for collagen detection in tissue reactions.. Histochem J..

